# Proprietary Medicines

**Published:** 1905-12

**Authors:** 


### Proprietary Medicines.

A good deal of pharisaical remarks are being published in certain journals against the advertisements in reputable medical journals of proprietary remedies in common use by the profession. We doubt if the tirades being made against them will materially influence the mass of the profession against the use of those preparations of well established reputation that have often been tried, their virtues not denied and ready to be tried again. The outbursts of some journals against such preparations has the appearance of "much ado about nothing." Nor are we prepared to appreciate the saint-like attitude of journals of the present day that attempt to uphold for professional criticism and rebuke some other leading journals because they admit into their advertising pages certain proprietary advertisements. There is a sort of "the temple of the Lord are we" censorship attempted that does not speak well for the record of some of the censuring journals. Until a year or so ago, even the *Journal of the American Medical Association,* as the representative one of the American profession, contained a number of such advertisements. But now that the membership subscription income alone is sufficient to run it, that journal turns upon its former patrons with a vim that is surprising, and reflects upon other reputable medicinal journals that do as it did.

Most of the proprietary remedies that have met with the approval of the profession at large have been analyzed—both qualitatively and quantitatively—and their compositions have been published in various prints. But rather than write prescriptions in detail for certain of these proprietary tablets or fluid preparations, and wait for the pharmacist to put them up, doctors generally, for the sake of readiness and general convenience, have preferred to prescribe the proprietary preparations direct—just as they might write for paregoric, or aromatic sulphuric acid, or vegetable cathartic pills—already prepared and on the shelf of the druggist. Yet, as to composition of these latter, which are in the U. S. Pharmacopeia, we venture little in assertion that relatively few practitioners who may be frequent prescribers of these articles can give their formula offhand. Squibb's diarrhea mixture, and many like preparations in constant use, are considered ethical, yet how many doctors can offhand write the formula for their preparation?

Of course there is an extreme view to be taken of this line of remark which we should not be considered as endorsing. The brazen quackishness of some advertisements of articles in public prints—their "King cure all" claims—expose them to very just censure. It is not always because of the real composition of these well known quack preparations, as revealed by the analytical chemists, that such preparations should be censured, as because of the false or fraudulent claims made regarding their remedial qualities, as published in newspapers, etc., simply to entrap the ignorant layman purchaser.

There is a middle ground between the extremes upon which it is safe for the profession to stand. Do not dispise all proprietary or ready-made preparations when their use seems indicated simply because their formula are not recalled at the moment they may be needed, provided sufficient is known of their active ingredients and of their average doses in given prescriptions; nor yet resort to those preparations which are typical of flagrant quackery.

In this connection, we should add our conviction that it would be better for the proprietary manufacturers themselves were they all to publish the active principals and proportions of their preparations in their professional advertisements; since it requires only a little analytical work on the part of chemists in almost every community to discover them. Such publications would not materially interfere with the prescription of their trade-marked preparations by doctors; and it would remove their preparations from that border line, one step beyond which they must be classed as absolutely unethical. While certain local druggists here and there might adopt like formula for their own trade, the established trade mark, as in the case of "lapactic pills," would well protect the original manufacturers. It would be hard for another manufacturer to "get the run" on these pills—so well established in use have they become—even should he adopt the exact proportions of the same drugs and attempt sale under another name.—*Virginia Medical Semi-Monthly.*

